# Exogenous Melatonin Enhances Cold Tolerance by Regulating the Expression of Photosynthetic Performance, Antioxidant System, and Related Genes in Cotton

**DOI:** 10.3390/plants13152010

**Published:** 2024-07-23

**Authors:** Jincheng Zhu, Hui Lou, Chen Yan, Wei Zhang, Zhibo Li

**Affiliations:** 1 The Key Laboratory of Oasis Eco-Agriculture, Agriculture College, Shihezi University, Shihezi 832000, China; zhujincheng@stu.shzu.edu.cn (J.Z.); louhui@stu.shzu.edu.cn (H.L.); yanchen@stu.shzu.edu.cn (C.Y.); 2 Biotechnology Research Institute, Xinjiang Academy of Agricultural and Reclamation Science, Shihezi 832000, China

**Keywords:** melatonin, antioxidant defense, cold stress, photosynthetic performance

## Abstract

In China, cotton is a significant cash crop, and cold stress negatively impacts the crop’s development, production, and quality formation. Recent studies have shown that melatonin (MT) can alleviate the damage to plants under cold stress and promote good growth and development. In this study, the morphological and physiological changes induced by exogenous melatonin pretreatment on ‘Xinluzao 33’ cotton seedlings under cold stress were examined to investigate its defensive effects. The results showed that 100 μM MT pretreatment improved the cold resistance of cotton most significantly. It also improved the wilting state of cotton under cold stress, greatly increased the photosynthetic rate (Pn), stomatal conductance (Gs), maximum photochemical efficiency (Fv/Fm), and photosynthetic performance index (PIabs) by 116.92%, 47.16%, 32.30%, and 50.22%, respectively, and mitigated the adverse effects of low-temperature. In addition, MT supplementation substantially reduced the accumulation of superoxide anion (O_2_^•−^) and hydrogen peroxide (H_2_O_2_) by 14.5% and 45.49%, respectively, in cold-stressed cotton leaves by modulating the antioxidant system, thereby mitigating oxidative damage. Furthermore, MT pretreatment increased the endogenous melatonin content (23.80%) and flavonoid content (21.44%) and considerably induced the expression of biosynthesis enzyme-related genes. The above results indicate that exogenous melatonin improves the low-temperature resistance of cotton seedlings by regulating photosynthetic performance, antioxidant enzyme activity, antioxidant content, endogenous melatonin and flavonoid content, and the expression levels of genes related to their synthesis.

## 1. Introduction

Plant growth and development are severely hampered by cold stress, which also restricts the range of locations where plants can be found in nature [1]. Reduced photosynthesis, altered gene expression levels, disruption of intracellular redox equilibrium, damage to membrane systems, and perturbation of basal metabolism are the principal impacts of low-temperature stress on plants [2]. Low temperature induces the production of reactive oxygen species (ROS) such as superoxide (O_2_^•−^), hydrogen peroxide (H_2_O_2_), and hydroxyl radicals (-OH). High concentrations of ROS can induce oxidative damage in plants; however, spatiotemporal transient accumulation of ROS, such as H_2_O_2_, is often used as a signal [3]. Plants have evolved a range of defense mechanisms, including enzymatic and non-enzymatic antioxidant systems, and ROS are partially scavenged by enzymes such as superoxide dismutase (SOD), peroxidase (POD), catalase (CAT), ascorbate peroxidase (APX), and glutathione peroxidase (GPX) [4]. Malondialdehyde (MDA), relative conductivity (REC), photosynthetic rate (Pn), stomatal conductance (Gs), maximum photochemical efficiency (Fv/Fm), and photosynthetic performance index (PIabs) can be used to assess the degree of damage and photosynthetic performance of plants under adversity [5]. An increase in MDA indicates damage to cell membranes, an increase in REC reflects an increase in cell membrane permeability, a decrease in Pn indicates a decrease in photosynthetic capacity, a decrease in GS affects photosynthesis, a decrease in Fv/Fm indicates a decrease in the efficiency of light energy conversion, and a decrease in PIabs reflects an overall impaired photosynthetic performance of plants [6]. The decrease in PIabs reflects the overall impaired photosynthetic performance of the plant. These indicators provide key information for understanding the response mechanism of plants to adversity. Previous studies have identified chalcone synthase (*CHS*), flavanone 3′-hydroxylase (*F3′H*), and flavanone 3-hydroxylase (*F3H*) as key enzymes in flavonoid biosynthesis. Chalconeisomerase (*CHI*) is a key enzyme in flavonoid metabolism. Dihydroflavonol-4- reductase (*DFR*) catalyzes the production of anthocyanin precursors from dihydroflavonols and flavanones [7]. Chlorophyllate a-oxygenase (*CAO*) is involved in the conversion of chlorophyll from a to b; Glutamic acid-1-semialdehyde transaminase (*HEML*) plays a key role in chlorophyll synthesis; Glutamyl t-RNA reductase (*HEMA*) catalyzes ALA synthesis and plays an important role in plant growth regulation; Pheide a oxygenase (*PAO*), 7-Hydroxymethyl Chlorophyll a reductase (*HCAR*), and Pheophytinase (*PPH*) are involved in chlorophyll degradation [8]. Under cold conditions, some cold-tolerant plants mainly exhibit elevated content of secondary metabolites, increased accumulation of osmoregulatory substances, enhanced antioxidant capacity, and up-regulation of cold resistance-related gene expression [9,10]. Most researchers have tried to enhance plant hardiness by screening and breeding new varieties, but in general, yield and quality are negatively correlated with adversity traits, and the long breeding period of new varieties makes it challenging to obtain desirable results in a short time. Therefore, there is an urgent need to find a method that is non-toxic, efficient, non-polluting, and effective in improving plant resistance.

Numerous scholars have successfully explored and applied various means, such as the exogenous application of phytohormones, osmoregulatory substances, gas molecules, and micronutrients, to significantly enhance the tolerance of plants to low-temperature stress [11,12,13]. Chang and colleagues discovered that the application of exogenous melatonin may mitigate the growth-inhibiting effects of low temperatures on barley seedlings. This was achieved by controlling the expression of the circadian clock genes *HvCCA1* and *HvTOC1* and the accumulation of several important physiological markers [14]. Zhang et al. found that melatonin improved cucumber cold tolerance by activating antioxidant enzymes and inducing key PSI, PSII-related genes, and carbon assimilation genes, and finally alleviated the damage to the photosynthetic apparatus of cucumber seedlings under cold stress [15]. Zhao et al. found that exogenous melatonin increased the expression of *CsZat12* and controlled the metabolism of pure amine (PA) and abscisic acid (ABA), thereby mitigating the damage caused by low-temperature stress in cucumbers [16].

Since it originated in the tropics and subtropics, cotton (*Gossypium* spp.) is susceptible to cold temperatures when it is growing and developing [17]. With the increasing demand, cotton cultivation areas have expanded to high latitudes and altitudes where the climate is unsuitable [18,19]. Therefore, the relevant planting areas are very prone to “reverse spring” weather, resulting in delayed growth and development, especially frequent cold damage during the budding and seedling stages, seriously affecting the final yield and quality of cotton [4,20], improving the cold tolerance of cotton seedlings is of great significance to increasing cotton yield.

There are currently studies available on the use of MT to increase resistance to verticillium wilt, heavy metal stress, drought, and salt [21,22,23,24], but little research has been performed on whether MT treatment regulates cold tolerance in cotton. Our focus was on studying growth situation, photosynthetic performance, the degree of cellular damage, and ROS homeostasis in cotton seedlings under cold stress by MT. Precisely, we also investigated the content of endogenous melatonin and flavonoids and their biosynthetic enzymes encoding the expression of related genes. This study attempted to provide a theoretical basis for MT to improve cold tolerance in cotton.

## 2. Results

### 2.1. 100 μM Exogenous Melatonin Alleviates Cold Stress Injury

**Figure 1:**
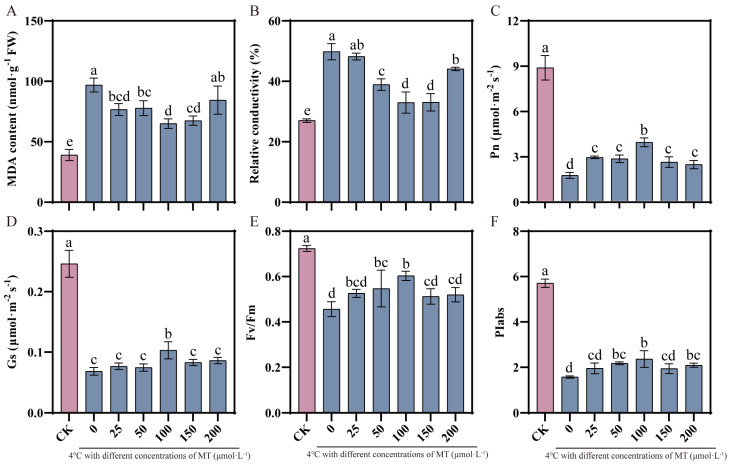
Effects of melatonin pretreatment at different concentrations on MDA. CK: under normal growth conditions after distilled water spray treatment (pink column). 0, 25, 50, 100, 150, 200: 4 °C with different concentrations of MT (0, 25, 50, 100, 150, 200 μmol·L^−1^) (pink column). MDA (**A**), REC (**B**), Pn (**C**), GS (**D**), Fv/Fm (**E**), and PIabs (**F**) in cotton under cold stress. Vertical bars in each column represent ±SD of three replicates, and different letters represent significant differences (*p* < 0.05).

Following low-temperature stress, cotton leaves were subjected to an analysis of the effects of varying doses of MT pretreatment on malondialdehyde (MDA), relative electrical conductivity, net photosynthetic rate, and stomatal conductance. The results showed that MDA content and relative conductivity (REC) in cotton leaves were drastically increased after cold stress by 148.28% and 84.52%, respectively (Figure 1A,B). However, Pn, Gs, Fv/Fm, and PIabs decreased markedly by 71.23%, 58.35%, 36.99%, and 72.42%, respectively (Figure 1C–F). Compared with spraying with 0 μM MT, spraying with 25–200 μM MT restored the Pn, Gs, Fv/Fm, and PIabs values to some extent, inhibited the accumulation of MDA, and eased the degree of cell damage. Among all MT treatments, 100 μM pretreatment had the best effect; Pn, Gs, Fv/Fm, and PIabs were greatly increased by 116.92%, 47.16%, 32.30%, and 50.22%, respectively, and MDA and REC were appreciably decreased by 34.09% and 33.79%, respectively, so this concentration was chosen for the follow-up test.

### 2.2. Effect of Exogenous Melatonin on Cotton Growth

**Figure 2:**
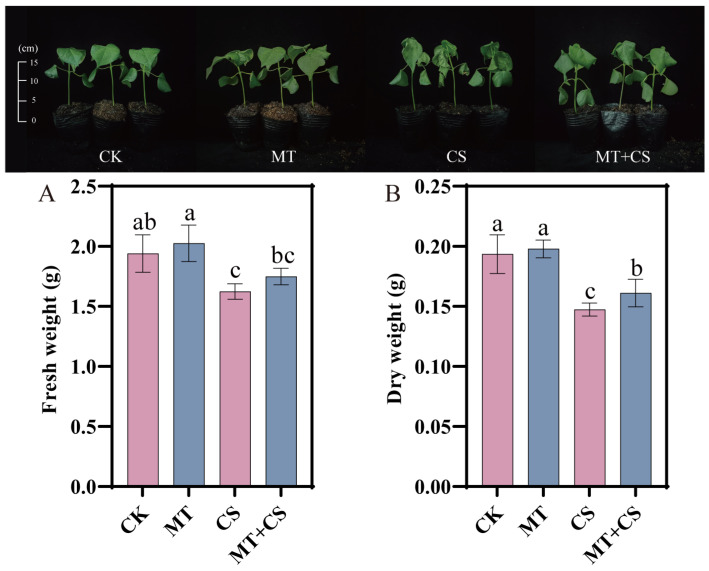
Effect of different treatments on the growth of cotton seedlings. CK: under normal growth conditions after distilled water spray treatment. MT: under normal growth conditions after 100 μM MT (the optimal concentration in the pre-screening) spray treatment. CS: spray distilled water treatment followed by 4 °C treatment for 24 h. MT + CS: spray 100 μM MT treatment followed by 4 °C treatment for 24 h. (**A**) Fresh weight, (**B**) Dry weight. Vertical bars in each column represent ±SD of three replicates, and different letters represent significant differences (*p* < 0.05).

At the end of the stress, both CS seedlings and MT + CS seedlings showed different degrees of leaf wilting, dehydration (Figure 2), and drooping, but the MT + CS group had markedly fewer symptoms than the CS group. After pretreatment with MT, growth limitations caused by CS stress were improved, and fewer reductions in fresh weight (7.69%) and dry weight (9.30%) were observed (Figure 2A,B). These findings imply that pretreating cotton seedlings with exogenous MT can lessen the harm that cold stress causes.

### 2.3. Effects of Exogenous Melatonin on the Antioxidant System

**Figure 3:**
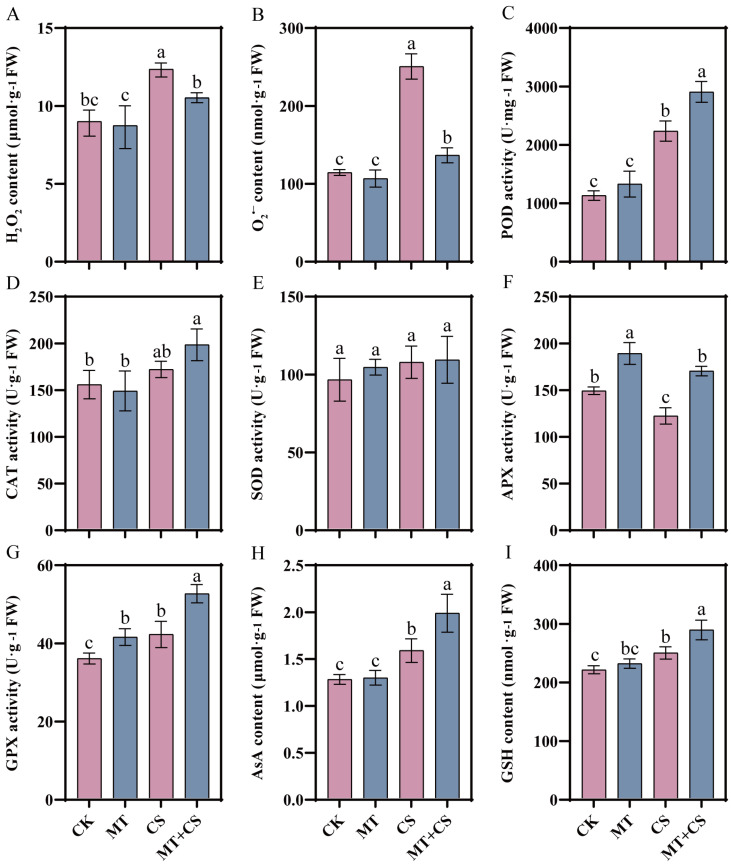
Effect of different treatments on H_2_O_2_ content. CK: under normal growth conditions after distilled water spray treatment. MT: under normal growth conditions after 100 μM MT (the optimal concentration in the pre-screening) spray treatment. CS: spray distilled water treatment followed by 4 °C treatment for 24 h. MT + CS: spray 100 μM MT treatment followed by 4 °C treatment for 24 h. H_2_O_2_ content (**A**), O_2_^•−^ content (**B**), antioxidant enzyme (POD (**C**), CAT (**D**), SOD (**E**), APX (**F**), GPX (**G**)) activities, and antioxidant (AsA (**H**), GSH (**I**)) content of cotton seedlings. Vertical bars in each column represent ±SD of three replicates, and different letters represent significant differences (*p* < 0.05).

Plants produce large amounts of ROS species as signaling molecules to deal with stress, but too much ROS can cause oxidative damage to themselves. Exogenous MT pretreatment under normal conditions did not change H_2_O_2_ and O_2_^•−^ content in leaves (Figure 3A,B). The H_2_O_2_ range and O_2_^•−^ in the CS group were substantially higher by 38.27% and 118.71%, respectively, as compared to the CK group. MT pretreatment may be able to lessen the harm produced by cold induction, as seen by the MT + CS group’s much lower values, which decreased by 14.50% and 45.49%, respectively, after the CS group.

In addition, we measured antioxidant enzyme (POD, CAT, SOD, APX, and GPX) activities (Figure 3C–G) and antioxidant (AsA and GSH) content (Figure 3H,I) in the different treatments. Compared with CK plants, the activities of POD and GPX enzymes in CS plants increased by 97.43% and 17.34%, respectively, while the activity of the APX enzyme decreased by 18.20% (Figure 3C–F). Compared with the CS plants, the activities of these five antioxidant enzymes were all increased to different degrees in the MT + CS plants, with POD, APX, and GPX activities notably increased by 29.96%, 39.78%, and 25.03%, respectively. AsA and GSH contents in MT + CS seedlings increased by 17.43% and 15.87%, respectively, and were substantially higher than those in CS seedlings.

### 2.4. Effects of Exogenous Melatonin on Photosynthetic Pigment Content

**Figure 4:**
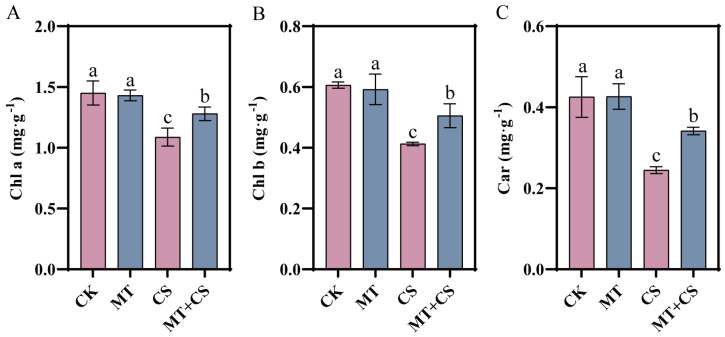
Effect of different treatments on chlorophyll a (**A**), chlorophyll b (**B**), and carotenoid (**C**) content of cotton seedlings. CK: under normal growth conditions after distilled water spray treatment. MT: under normal growth conditions after 100 μM MT (the optimal concentration in the pre-screening) spray treatment. CS: spray distilled water treatment followed by 4 °C treatment for 24 h. MT + CS: spray 100 μM MT treatment followed by 4 °C treatment for 24 h. Vertical bars in each column represent ±SD of three replicates, and different letters represent significant differences (*p* < 0.05).

Photosynthesis is carried out with the participation of photosynthetic pigments, trapping light radiation and converting it into CO_2_^−^ assimilated energy. According to Figure 4, compared with the CK seedlings, chlorophyll a, chlorophyll b, and carotenoid in CS seedlings decreased markedly by 25.10%, 31.90%, and 42.50%, respectively. On the contrary, the chlorophyll a, b, and carotenoid contents of MT + CS seedlings were reduced by 11.8%, 16.6%, and 19.7%, respectively, compared with CK seedlings, which was a significantly smaller decrease.

### 2.5. Effects of Exogenous Melatonin on Flavonoid Content and Biosynthesis Enzyme-Related Genes Expression

**Figure 5:**
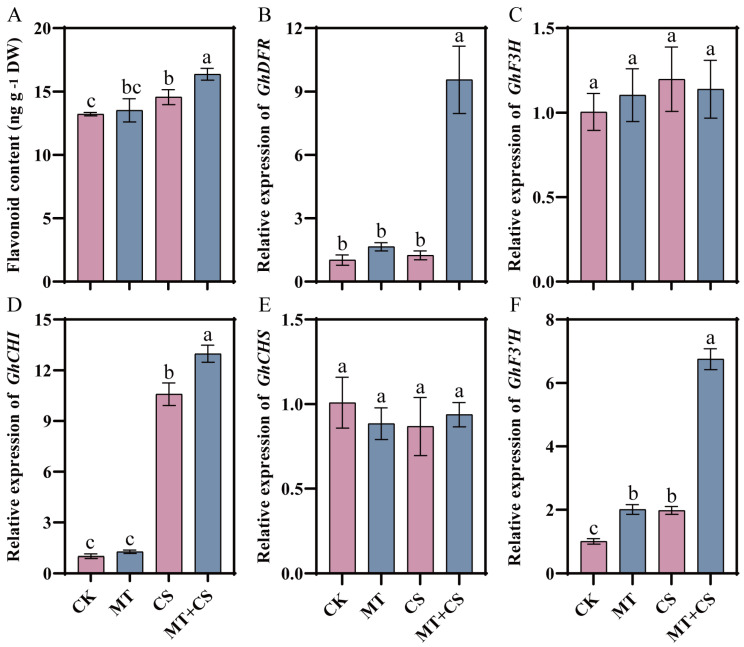
Effect of different treatments on flavonoid content (**A**) and flavonoid biosynthesis enzyme-related gene (*GhDFR* (**B**), *GhF3H* (**C**), *GhCHI* (**D**), *GhCHS* (**E**), *GhF3′H* (**F**)) expression in cotton seedlings. CK: under normal growth conditions after distilled water spray treatment. MT: under normal growth conditions after 100 μM MT (the optimal concentration in the pre-screening) spray treatment. CS: spray distilled water treatment followed by 4 °C treatment for 24 h. MT + CS: spray 100 μM MT treatment followed by 4 °C treatment for 24 h. Vertical bars in each column represent ±SD of three replicates, and different letters represent significant differences (*p* < 0.05).

One of the most significant and prevalent secondary metabolites in plants, flavonoids can improve the plant’s ability to withstand stress and produce antioxidants naturally. In the present study, the flavonoid content in the MT group was slightly higher than that in the CK group, but the difference was not significant. Compared with the CK group, the flavonoid content in the CS and MT + CS groups increased by 10.20% and 23.80%, respectively (Figure 5).

The expression levels of flavonoid synthesis genes *GhDFR*, *GhF3H*, *GhCHI*, and *GhF3′H* were up-regulated to varying degrees in the MT plants compared to the CK plants, and only *GhCHS* was slightly down-regulated, but there was no significant difference. Compared with CK seedlings, the expression levels of *GhDFR*, *GhF3H*, *GhCHI*, and *GhF3′H* were up-regulated to varying degrees in MT + CS seedlings, among which *GhDFR*, *GhCHI*, and *GhF3′H* were considerably up-regulated, increasing by 671.58%, 22.65% and 241.29%, respectively.

### 2.6. Effects of Exogenous Melatonin on Endogenous Melatonin Content and Synthesis-Related Genes Expression

**Figure 6:**
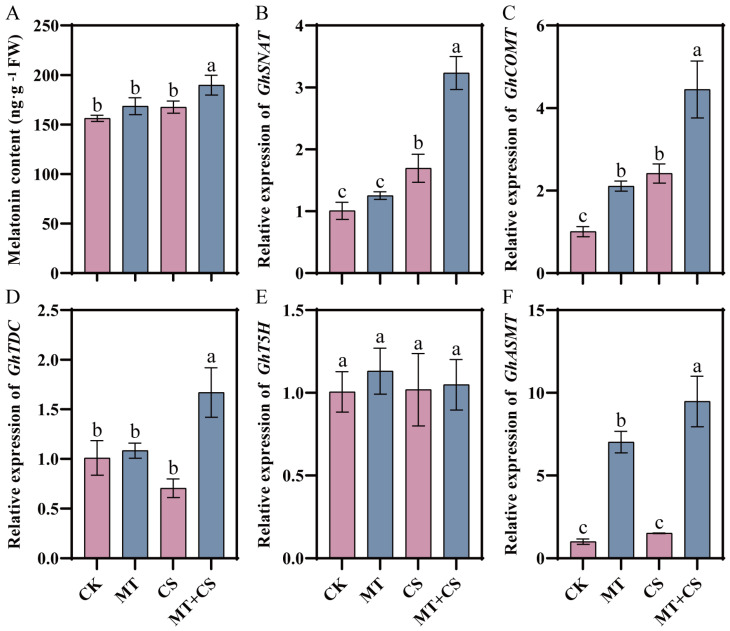
Effects of different treatments on endogenous melatonin content (**A**) and melatonin biosynthesis enzyme-related gene (*GhSNAT* (**B**), *GhCOMT* (**C**), *GhTDC* (**D**), *GhT5H* (**E**), *GhASMT* (**F**)) expression in cotton seedlings. CK: under normal growth conditions after distilled water spray treatment. MT: under normal growth conditions after 100 μM MT (the optimal concentration in the pre-screening) spray treatment. CS: spray distilled water treatment followed by 4 °C treatment for 24 h. MT + CS: spray 100 μM MT treatment followed by 4 °C treatment for 24 h. Vertical bars in each column represent ±SD of three replicates, and different letters represent significant differences (*p* < 0.05).

Endogenous melatonin content was increased by 21.44% in MT + CS plants compared to CK plants (Figure 6). The expression of *GhSNAT* and *GhCOMT* was up-regulated in the CS group compared to the CK group, increasing by 68.44% and 240.14%, respectively, while the expression of *GhTDC* was down-regulated compared to the CK group, decreasing by 30.19%. The gene expression levels of melatonin biosynthesis genes *GhSNAT*, *GhCOMT*, *GhTDC*, and *GhASMT* in the MT + CS group were up-regulated to varying degrees compared with those in the CK group, among which *GhCOMT* and *GhASMT* were greatly up-regulated by 109.61% and 595.61%, respectively. 

### 2.7. Effects of Exogenous Melatonin on the Expression of Genes Related to Chlorophyll Degradation and Synthesis in Cotton under Low-Temperature Stress

**Figure 7:**
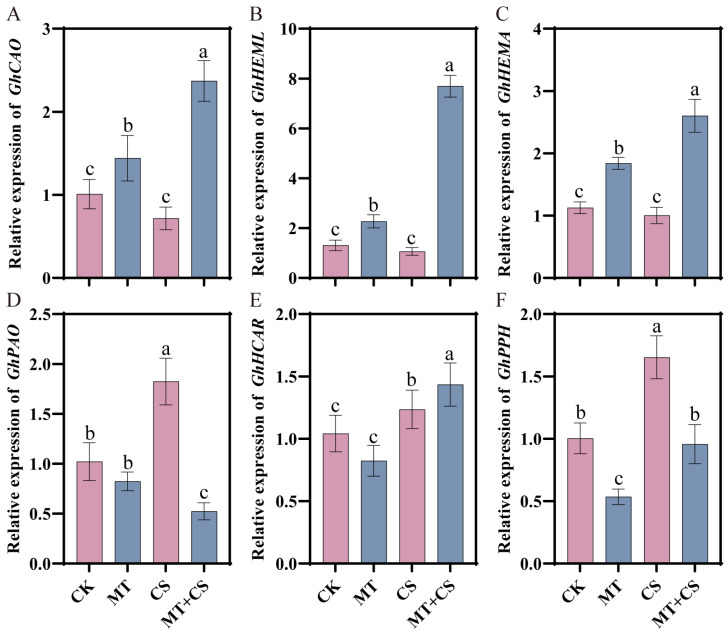
Effects of different treatments on the expression of genes related to chlorophyll degradation and synthesis in cotton (*GhCAO* (**A**), *GhHEML* (**B**), *GhHEMA* (**C**), *GhPAO* (**D**), *GhHCAR* (**E**), *GhPPH* (**F**)). CK: under normal growth conditions after distilled water spray treatment. MT: under normal growth conditions after 100 μM MT (the optimal concentration in the pre-screening) spray treatment. CS: spray distilled water treatment followed by 4 °C treatment for 24 h. MT + CS: spray 100 μM MT treatment followed by 4 °C treatment for 24 h. Vertical bars in each column represent ±SD of three replicates, and different letters represent significant differences (*p* < 0.05).

Chlorophylls are a class of green pigments contained in photosynthesizing organisms and are magnesium porphyrin compounds that belong to a family of lipid-containing pigments. According to Figure 7, the expression levels of chlorophyll synthesis genes *GhCAO*, *GhHEML*, and *GhHEMA* were apparently not dramatically different in the CS group compared with the CK group. However, the expression of *GhCAO*, *GhHEML,* and *GhHEMA* were remarkably up-regulated in the MT + CS group, which increased by 135.06%, 488.47%, and 131.02%, respectively. The expression levels of chlorophyll-degrading genes *GhPAO*, *GhHCAR*, and *GhPPH* were appreciably up-regulated in the CS group compared with the CK group, which increased by 78.67%, 18.54%, and 64.79%, respectively. Meanwhile, the expression of *GhPAO* was significantly down-regulated in the MT + CS group, with a reduction of 48.71%. Among them, the *GhPPH* gene expression was not different from that of the MT group. 

### 2.8. Correlation Analysis of Cotton Antioxidant System with Flavonoids and Melatonin

**Figure 8:**
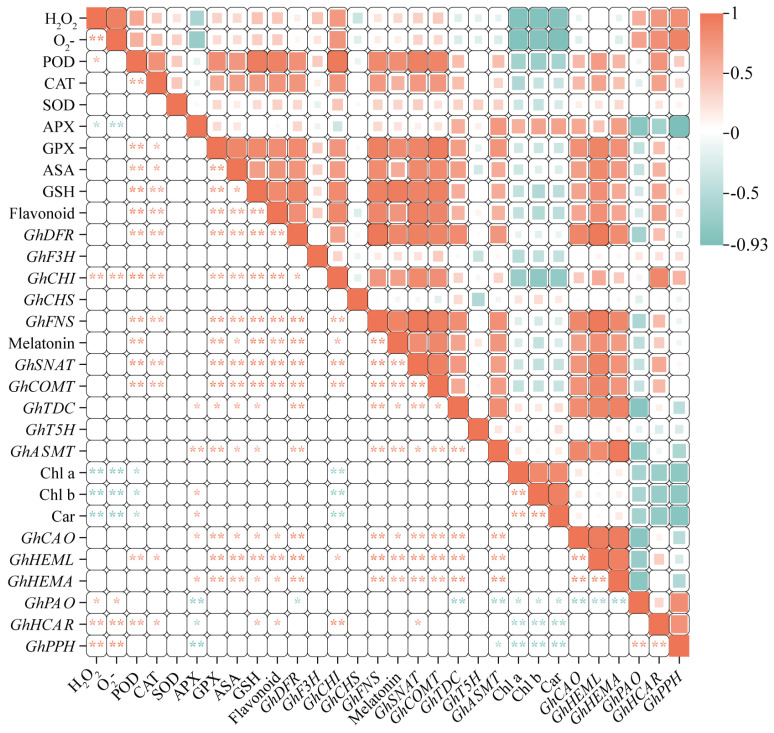
Correlation analysis of cotton antioxidant system with flavonoids and melatonin. (Different size of colored cells map the magnitude of correlation,* *p* < 0.05; ** *p* < 0.01).

Through a comprehensive analysis of the correlation of antioxidant system parameters with flavonoids and melatonin in cotton, the antioxidant system parameter POD showed a highly significant positive correlation with flavonoid content and its synthesized genes, *GhDFR*, *GhCHI*, and *GhFNS*. Meanwhile, there was a highly significant positive correlation between melatonin content and its synthesizing genes, *GhSNAT* and *GhCOMT*, and a significant negative correlation with Chl a, Chl b, and Car (Figure 8).

## 3. Discussion

Cold stress greatly affects the yield of cotton by disrupting its physiological metabolism and its morphological structure and stunting its growth [25]. Previous studies have found that MT, a ubiquitous multifunctional signaling molecule, can improve plant tolerance to low-temperature stress by regulating physiological and biochemical processes [11]. This study showed that the leaves of cotton seedlings exhibited significant wilting and crinkling after cold stress treatment, and MT pretreatment could reduce the damage caused by the stress (Figure 2). Similar findings were also verified in banana seedlings, pepper seedlings, and strawberry seedlings under cold stress [26,27,28].

In plants, photosynthesis is a vital physiological mechanism that produces 95% of the dry matter accumulation in plants [29]. The chlorophyll content is crucial for the smooth progress of photosynthesis, and its level determines the rate of photosynthesis, which directly affects plant growth and yield [30,31,32]. In this study, we found that the net photosynthetic rate, stomatal conductance, and chlorophyll content of cotton seedlings were considerably decreased under cold stress, but MT pretreatment alleviated the degradation of photosynthetic pigments caused by cold stress, indicating that MT can effectively promote the synthesis of photosynthetic pigments under stress conditions (Figure 4). According to earlier research, exogenous MT dramatically up-regulates gene expression linked to chlorophyll production and down-regulates gene expression related to chlorophyll breakdown, hence maintaining chlorophyll concentration under stress [33,34,35]. Furthermore, chlorophyll fluorescence measurements are thought of as internal sensors for researching the connection between plant photosynthesis and the environment. To a certain extent, these metrics can accurately indicate a plant’s ability to withstand cold temperatures [36,37]. Studies have shown that Fv/Fm is relatively stable when plants are not under stress, but it will be markedly reduced when plants are under pressure or mechanical damage [38]. The present study revealed that low-temperature stress significantly reduced the Fv/Fm values of cotton leaves, which is a clear indication of the inhibition of the photosynthetic system. However, it is noteworthy that the Fv/Fm values of the optimal melatonin pretreated group were significantly elevated compared with those of the untreated group, a finding that suggests that the application of exogenous melatonin effectively mitigated the inhibition of the photosynthetic capacity of cotton by low-temperature stress (Figure 1E). In addition, our study also found that the PIabs values of the melatonin-pretreated group were significantly higher than those of the untreated group under low-temperature conditions (Figure 1F), which coincided with the results of a study on bananas conducted by Liu et al. [26] and further confirmed the positive role of melatonin in enhancing plant stress tolerance.

Cold stress disrupts the plant’s own ROS scavenging mechanism, causing excessive ROS accumulation and lipid peroxidation, which affects plant growth and development [39]. MT directly scavenges ROS to act as an antioxidant and lessen damage to plants [40]. It was shown that in order to keep the balance between ROS generation and scavenging, a single MT molecule may interact with eight or more ROS molecules continually [41]. The MT congener 5-methoxyindole and the downstream product of MT metabolism, N1-acetyl-N2-formyl-5-methoxykynura-mine, were also found to have different degrees of antioxidant activity [42,43,44]. According to earlier research, MT supplementation under stressful circumstances boosted the expression of genes encoding related synthetic enzymes and stimulated the manufacture of endogenous MT [45]. In this study, we observed that the endogenous melatonin (MT) content and the expression levels of its biosynthesis genes (*GhSNAT*, *GhCOMT*, *GhTDC*, *GhASMT*) were up-regulated to different degrees in cotton seedlings after cold stress. It is noteworthy that these up-regulation phenomena showed more significance when cotton seedlings were pretreated with MT (Figure 6). Accordingly, we hypothesized that cotton seedlings would actively increase the level of melatonin in response to cold stress, and MT pretreatment could accelerate the process of melatonin synthesis, thus scavenging reactive oxygen species (ROS) more efficiently and mitigating the damage caused by cold stress. This finding provides a new perspective for understanding how cotton seedlings respond and adapt to cold stress.

Important components of the antioxidant defense system include both non-enzymatic and enzyme-based antioxidants [46]. According to earlier research, MT pretreatment can lessen the harm that cold stress causes to oilseed rape and watermelon by increasing the activity of antioxidant enzymes and the expression of genes encoding them [47,48]. MT pretreatment significantly enhanced the activities of antioxidant enzymes, including SOD, CAT, POD, APX, and GPX, as well as increased the contents of antioxidants AsA and GSH in cotton under stress conditions (Figure 3). These results fully demonstrated that MT pretreatment had a positive effect on enhancing the performance of the cotton antioxidant system and could maintain the balance between ROS production and accumulation more effectively. These findings further validate the hypothesis that MT scavenges ROS directly or indirectly through the activation of antioxidant enzymes and fit with previous research results [49].

It has been shown that by taking part in the manufacture of secondary metabolites in plants, MT increases tolerance to a range of stressors. A significant portion of the phenylpropane metabolic system, flavonoid molecules play a major role in the growth, development, and stress tolerance of plants [50,51]. Previous studies have found that the content of flavonoids in Arabidopsis leaves is positively correlated with cold tolerance, proving that flavonoids are one of the key factors determining cold acclimation and freezing tolerance of Arabidopsis [52,53,54]. Sun et al. found that exogenous melatonin increased the expression of *CcF3′h-5* through the transcription factor *CcPCL1*, promoted the accumulation of luteolin and its derivatives, and speculated that flavonoids could be used as essential mediators in melatonin to enhance the tolerance of plants to various stresses [55]. In the present study, it was found that there was a significant increase in flavonoid content in cotton leaves under low temperature stress conditions. However, when cotton was pretreated with melatonin, the increase in flavonoid content was more significant. Meanwhile, we observed that the up-regulated expression of key genes in the flavonoid synthesis pathway (*GhDFR*, *GhCHI*, and *GhF3′H*) was more prominent after melatonin pretreatment (Figure 5), and this finding provides a new perspective for an in-depth understanding of flavonoid biosynthesis and regulatory mechanisms. Therefore, we believe that MT pretreatment enhanced the resistance of cotton to cold stress can be reflected by the increase in flavonoid content and biosynthetic gene expression levels to be regulated.

## 4. Materials and Methods

### 4.1. Effects of Exogenous Melatonin on Photosynthetic Pigment Content

The experimental material ‘Xinluzao 33’ was purified, preserved, and provided by the Cotton Molecular Breeding Laboratory of Oasis Ecology Laboratory of Shihezi University. After choosing the seeds with whole granules, they were steeped in 75% alcohol for 10 min and then rinsed 3–4 times in ultrapure water. The sterilized seeds were soaked in 50 °C–60 °C warm water for 20 min and then placed in a germination box with sterilized gauze. After being accelerated to sprout at 25 °C in the dark for 48 h (keeping the gauze moist all the time), the germinated seeds were planted in a nutrient bowl and cultured in an artificial intelligence climate box (ZDN-1000g-2, Ningbo, China). The culture substrate was peat: vermiculite = 3:1, and the climate box conditions were set as follows: the temperature was 28 °C/25 °C (day/night), relative humidity was 60%, light intensity was 32,000 Lux, and photoperiod was 16 h/8 h (day/night).

After cotton seedlings grew to ‘three leaves in one heart,’ different concentrations (0, 25, 50, 100, 150, and 200 μM) of MT (CAS: 73-31-4, Sigma, St. Louis, MO, USA) were applied. The solution required uniform spraying and spraying to the leaf surface as the standard, once in the morning and once in the evening (9 a.m. and 9 p.m.), with continuous spraying for three days while avoiding light after spraying. Each treatment was applied to 20 seedlings. After the last spraying treatment, the cotton was allowed to stand for 24 h in preparation for low-temperature stress treatment. The procedure was performed as follows: cotton was placed in a climate chamber with a temperature of 4 °C/4 °C (day/night), relative humidity of 60%, a light intensity of 32,000 Lux, and photoperiod of 16 h/8 h (day/night) for 24 h. After the stress, the gas exchange measurements, fast chlorophyll fluorescence parameters, malondialdehyde content, and electrical conductivity of the third genuine leaf of cotton seedlings were measured to screen the optimal melatonin concentration for improving the cold tolerance of cotton seedlings.

To cultivate new cotton seedlings, seed disinfection, germination promotion, and seedling cultivation were the same as above. When cotton seedlings reached the “three leaves and one heart” stage, they were divided into two groups. One group was treated with 100 μM MT, and the other group was treated with distilled water (CK), with the same treatment method as above. The seedlings of each group were placed in incubations at 28 °C/25 °C (day/night) and 4 °C/4 °C (day/night) for 24 h (CK and MT seedlings treated with 4 °C low-temperature were named CS and MT + CS, respectively). Thirty cotton seedlings were collected from each treatment. The third true leaves of cotton seedlings under different treatments were taken for the determination of physiological indicators and gene expression.

### 4.2. Measurement of Photosynthetic Parameters

Photosynthetic parameters, including Pn (net photosynthetic rate) and Gs (stomatal conductance), were assayed (9 to 11 a.m.) by LI-COR 6400 Photosynthesis System (Li-Cor, Lincoln, NE, USA). The light source was the instrument’s red and blue built-in light source, and the light intensity was set at 1000 μmol·m^−2^·s^−1^. The maximum photochemical efficiency of PSII (Fv/Fm) and light energy absorption performance index (PIabs) were measured by a portable pulse adjustment fluorometer (Handy PEA, Hansatech, UK). The leaves were fully dark-adapted for 30 min before the assay and then induced using 3000 μmol·m^−2^·s^−1^ red light for 2 s. Seedlings with the same growth rate were selected from the same leaf position for each treatment, and five biological replicates were set up. Acetone extraction was used to measure the amount of chlorophyll. The UV1800 Spectrophotometer was used to measure the absorbance at 645, 663, and 440 nm, respectively, to compute the amounts of chlorophyll a (Chl a), chlorophyll b (Chl b), and carotenoid (Caro) [56].

### 4.3. Measurement of Malondialdehyde Content and Relative Conductivity

In a test tube, 0.4 g of leaves were mixed with 10 mL of distilled water, and the vacuum was evacuated for 20 min. A conductivity meter (DDSJ-308A, Shanghai Kanglu Instrument Equipment Co., Ltd., Shanghai, China) was used to measure the mixture’s initial conductivity (S1). After incubating in a boiling water bath for 10 min, the mixture was cooled and its conductivity was measured (S2). A conductivity test was also conducted on the distilled water (S0). Electrolyte leakage (%) = [(S1 − S0)/(S2 − S0)] × 100 [57]. Using the thiobarbituric acid method, malondialdehyde (MDA) content was calculated from absorption values at 450, 532, and 600 nm [58], and three independent replications were conducted.

### 4.4. Measurement of H_2_O_2_ and O_2_^•−^ Content, Antioxidant Enzyme Activity, and Antioxidant Content

The contents of H_2_O_2_, O_2_^•−^, ascorbic acid (AsA), reduced glutathione (GSH), and the activities of peroxidase (POD), catalase (CAT), superoxide dismutase (SOD), ascorbic acid peroxidase (APX), and glutathione peroxidase (GPX) were measured by using the kit on a UV-1800 spectrophotometer. APX) and glutathione peroxidase (GPX) activities. These kits (H_2_O_2_-2-Y, SA-2-G, ASA-2-W, GSH-2-W, POD-2-Y, CAT-2-Y, SOD-2-Y, APX-2-W, GPX-2-W, Suzhou, China) were produced by Suzhou Comin Biotechnology Co.

H_2_O_2_ forms a complex with titanium sulfate with characteristic absorption at 415 nm. O_2_^•−^ reacts with hydroxylamine hydrochloride to form NO_2_^−^, and in the presence of p-aminobenzenesulfonic acid and α-naphthylamine, an azo compound is formed with a characteristic absorption peak at 530 nm. In acetic acid solution, ASA reacts with the solid blue salt B to form a yellow derivative of oxalylhydrazide-2-hydroxybutyrolactone, the absorbance of which is measured at a maximum absorption wavelength of 420 nm. GSH reacts with DTNB to form a complex with a characteristic absorption peak at 412 nm. POD catalyzes the oxidation of specific substrates by H_2_O_2_ with characteristic light absorption at 470 nm. CAT is able to decompose H_2_O_2_ so that the absorbance of the reaction solution at 240 nm decreases with reaction time, and CAT activity can be calculated from the rate of change of absorbance. The SOD activity was determined by the NBT method, i.e., the reduced riboflavin is highly susceptible to re-oxidation and generates superoxide anion radicals, and reduces NBT to methanone, with a characteristic absorption peak at 560 nm. APX catalyzed the reduction of H_2_O_2_ by AsA, and the activity of APX could be calculated by measuring the amount of AsA oxidized. GPX catalyzes the peroxide oxidation of GSH to GSSG, and glutathione reductase uses NADPH to reduce GSSG and regenerate GSH, which has a characteristic absorption peak at 340 nm, and the rate of reduction of light absorption in this band was measured to calculate GPX activity. The procedure is described in the instructions (Supplementary Materials S2), and three biological replicates were used for each indicator.

### 4.5. Measurement of Endogenous Melatonin Content and Flavonoid Content

Melatonin content was determined by plant melatonin enzyme-linked immunosorbent assay analytical kit (96T/48T, Shanghai, China). The 0.1 g sample was mashed with normal saline and centrifuged at 3000 rpm for 10 min. A Plant MT ELISA Kit was used to detect melatonin. Using the kit of Suzhou Comin Biotechnology Co., Ltd. (LHT-2-G, Suzhou, China), flavonoid content was determined by measuring absorbance values at 510 nm. A total of three biological replicates were tested on each sample.

### 4.6. Real-Time Polymerase Chain Reaction Analysis of Gene Expression

EasyPure Plant RNA Kit was used to extract total RNA, and EasyScript One-Step gDNA Removal and cDNA Synthesis SuperMix kit was used to reverse transcribe it into cDNA. SYBR Green was used in qRT-PCR reactions on a Light Cycler 480II sequence detection system, following the method of Xiong et al. [59]. *GhUBQ7* (DQ116441) was used as the internal control since it is expressed stably in cotton plants and is unaffected by treatment or genotype. Suitable primers were designed using Primer Premier 5.0 software (Table S1), and relative expression was calculated using the 2^−ΔΔCt^ method [60].

### 4.7. Statistical Analysis

Statistical analysis of all data was performed by SPSS 24.0 (IBM, Armonk, NY, USA) software with single factor variance (ANOVA). The least significant difference method (LSD) was used for multiple comparisons. Statistical significance was defined as *p* < 0.05. The error bars in all graphs represent the standard error of the mean.

## 5. Conclusions

In this research, we examined the notable decline in resistance of cotton seedlings to cold stress due to impaired leaf dehydration, inhibited photosynthetic performance, excessive accumulation of ROS, and increased degree of lipid peroxidation. In contrast, MT treatment increased the resistance of cotton seedlings to cold stress. MT pretreatment enhanced the antioxidant defense system of cotton seedlings and delayed leaf wilting under low-temperature stress, thus enhancing photosynthetic performance. In addition, MT pretreatment resisted cold stress by affecting endogenous melatonin content, flavonoid content, and biosynthesis-related genes’ expression. In conclusion, exogenous melatonin supplementation effectively sustained photosynthesis, ROS scavenging, and redox mechanisms in cotton under cold stress, thereby mitigating damage to the seedlings.

## Data Availability

Data is contained within the article and Supplementary Materials.

## Supplementary Materials

The following supporting information can be downloaded at https://www.mdpi.com/article/10.3390/plants13152010/s1, Table S1: List of primers. S2: Kit Instruction Manual.
