# Supplementary material for: Exogenous Melatonin Enhances Cold Tolerance by Regulating the Expression of Photosynthetic Performance, Antioxidant System, and Related Genes in Cotton
Source: Plants (Basel). 2024 Jul 23;13(15):2010. doi: 10.3390/plants13152010 (PMC11314530; doi:10.3390/plants13152010)
Supplement: Supplementary file 1 [file plants-13-02010-s001.zip › S2 Kit Instruction Manual .pdf]

Hydrogen Peroxide (H_2O_2) Assay Kit Instructions

Micro Method 100 Tubes/96 Samples

Before the formal determination, be sure to take 2-3 samples with expected large differences for pre-determination.

Determination Significance:

H_2O_2 is the most common reactive oxygen molecule in organisms, mainly catalyzed by SOD and XOD, and degraded by CAT and POD, etc. H_2O_2 is not only an important reactive oxygen species but also a hub for the interconversion of reactive oxygen. On the one hand, H_2O_2 can directly or indirectly oxidize intracellular nucleic acids, proteins and other large biomolecules, and damage the cell membrane, thereby accelerating cell aging and disintegration; on the other hand, H_2O_2 is also a key regulatory factor in many oxidative emergency reactions.

Principle of Determination:

H_2O_2 reacts with titanium sulfate to form a yellow peroxide-titanium complex, which has a characteristic absorption at 415nm.

Instruments and Supplies to be Prepared:

Visible spectrophotometer/enzyme marker, desktop centrifuge, adjustable pipette, micro quartz cuvette/96-well plate, acetone 100mL, concentrated hydrochloric acid 5mL, mortar and ice.

Composition and Preparation of Reagents:

Reagent 1: Acetone 100mL × 1 bottle, store at 4°C; (self-prepared)

Reagent 2: Powder × 1 bottle, store at 4°C; add 3mL concentrated hydrochloric acid before use and dissolve thoroughly. (Dissolving time is relatively long, about 30 minutes, can be heated at 40-60°C, be sure to prepare in advance)

Reagent 3: Liquid 6.5mL × 1 bottle, store at 4°C;

Reagent 4: Liquid 30mL × 1 bottle, store at 4°C;

H_2O_2 Extraction:

1. Preparation of bacterial or cell samples: Collect bacteria or cells into a centrifuge tube, centrifuge and discard the supernatant; according to the ratio of the number of bacteria or cells (104) to the volume of Reagent 1 (mL) is 500~1000:1 (it is recommended to add 1mL of Reagent 1 to 500 million bacteria or cells), ultrasonicate to break the bacteria or cells (ice bath, power 20% or 200W, ultrasonic for 3 seconds, interval for 10 seconds, repeat 30 times); adjust the volume to 1mL with Reagent 1; centrifuge at 8000g for 10 minutes at 4°C, take the supernatant and keep it on ice for testing.
2. Preparation of tissue samples: According to the ratio of tissue mass (g) to the volume of Reagent 1 (mL) is 1:5~10 (it is recommended to take about 0.1g of tissue, add 1mL of Reagent 1) for homogenization in an ice bath; transfer to an EP tube, adjust the volume to 1mL with Reagent 1, centrifuge at 8000g for 10 minutes at 4°C, take the supernatant and keep it on ice for testing.
3. Serum (plasma) samples: Directly detect.

Determination Steps:

1. Preheat the spectrophotometer or enzyme marker for more than 30 minutes, set the

wavelength to 415nm, and zero with distilled water.
2. Heat Reagents 2, 3, and 4 at 37°C (mammals) or 25°C (other species) in a water bath for more than 10 minutes.
3. In the EP tube, add the following reagents in order:

Reagent Name (μL)	Test	Control
Sample	250	
Reagent 1		250
Reagent 2	25	25
Reagent 3	50	50
Centrifuge at 4000g, 25°C for 10 minutes, discard the supernatant, and keep the precipitate.		
Reagent 4	250	250

After adding Reagent 4 to dissolve the precipitate, let it stand at room temperature for 5 minutes, take 200μL and transfer to a micro quartz cuvette or 96-well plate to measure the absorbance A at 415nm. The control tube only needs to be done once. Calculate ΔA = A Test - A Control.

Precautions:

1. Since Reagent 1 is volatile, it must be precooled before adding, and grinding must be done on ice.
2. The volatility of the reagents in this kit is high, please wear disposable gloves and a mask.

H_2O_2 Content Calculation:

a. Calculation formula for determination with micro quartz cuvette:

1. Regression curve under standard conditions: y = 0.7488x + 0.0006 (x is the standard concentration, μmol/mL; y is ΔA).
2. Calculation of H_2O_2 content in serum (plasma): H_2O_2 content (μmol/mL) = (ΔA - 0.0006) ÷ 0.7488 = 1.34 × (ΔA - 0.0006)
3. Calculation of H_2O_2 content in bacteria, cells, or animal tissues (1) Calculate H_2O_2 content according to protein concentration (μmol/mg prot) = [(ΔA - 0.0006) ÷ 0.7488 × V1] ÷ (V1 × Cpr) = 1.34 × (ΔA - 0.0006) ÷ Cpr. It needs to be measured separately, it is recommended to use our BCA protein content assay kit. (2) Calculate H_2O_2 content according to sample mass (μmol/g fresh weight) = [(ΔA - 0.0006) ÷ 0.7488 × V1] ÷ (W × V1 ÷ V2) = 1.34 × (ΔA - 0.0006) ÷ W

(3) Calculate according to the density of bacteria or cells:
H_2O_2 content (μmol/104) = [(ΔA - 0.0006) ÷ 0.7488 × V1] ÷ (500 × V1 ÷ V2) = 0.0027 × (ΔA - 0.0006)

V1: Volume of sample added to the reaction system, 0.25mL; V2: Volume of extraction liquid added, 1mL; Cpr: Sample protein concentration, mg/mL; W: Sample mass, g; 500: Total number of cells or bacteria, 5 million.

b. Calculation formula for determination with 96-well plate:

1. Regression curve under standard conditions: y = 0.3744x + 0.0006 (x is the standard concentration, μmol/mL; y is ΔA).
2. Calculation of H_2O_2 content in serum (plasma): H_2O_2 content (μmol/mL) = (ΔA - 0.0006) ÷ 0.3744 = 2.67 × (ΔA - 0.0006)

3. Calculation of H_2O_2 content in bacteria, cells, or tissues

(1) Calculate H_2O_2 content according to protein concentration (μmol/mg prot) = [(ΔA - 0.0006) ÷ 0.3744 × V1] ÷ (V1 × Cpr) = 2.67 × (ΔA - 0.0006) ÷ Cpr. It needs to be measured separately, it is recommended to use our BCA protein content assay kit.

(2) Calculate H_2O_2 content according to sample mass (μmol/g fresh weight) = [(ΔA - 0.0006) ÷ 0.3744 × V1] ÷ (W × V1 ÷ V2) = 2.67 × (ΔA - 0.0006) ÷ W

(3) Calculate according to the density of bacteria or cells:

H_2O_2 content (μmol/104) = [(ΔA - 0.0006) ÷ 0.3744 × V1] ÷ (500 × V1 ÷ V2) = 0.0054 × (ΔA - 0.0006)

Note: Scale curvature range is 0.1μmol/mL-2μmol/mL, absorbance ΔA linear range is 0.03-1, if ΔA is more than 1, then need to be diluted, the calculation formula multiplied by the corresponding dilution times.

Superoxide Anion (Superoxide anion, OFR) Assay Kit Instructions

Spectrophotometric Method 50 Tubes/48 Samples

Note: Select 2-3 samples with expected large differences for pre-determination before the formal test.

Significance of Determination:

Superoxide anions and other reactive oxygen species in organisms have roles in immunity and signal transduction, but excessive accumulation can damage cell membranes and biological macromolecules, leading to metabolic abnormalities in body cells and tissues, and thus causing various diseases.

Principle of Determination:

Superoxide anions react with hydroxylamine hydrochloride to form NO_2^-. Under the action of sulfanilic acid and α-naphthylamine, NO_2^- generates a red azo compound with a characteristic absorption peak at 530nm. The content of O_2^- in the sample can be calculated based on the ΔA value. The reaction equation is $NH_2OH + 2O_2- + H^+ \rightarrow NO_2^- + H_2O_2 + H_2O$.

Experimental Supplies and Equipment to be Prepared:

Balance, water bath, centrifuge, visible spectrophotometer, 1 mL glass cuvette, chloroform, and distilled water.

Composition and Preparation of Reagents:

Extraction liquid: Liquid 100mL × 1 bottle, store at 4°C.

Reagent one: Liquid 25mL × 1 bottle, store at 4°C.

Reagent two: Liquid 20mL × 1 bottle, store at 4°C in the dark.

Reagent three: Liquid 20mL × 1 bottle, store at 4°C in the dark.

Reagent four: Chloroform, self-prepared.

Extraction of Superoxide Anions:

1. Plant, animal tissues: Homogenize on ice at a ratio of tissue mass (g) to extraction liquid volume (mL) of 1:5~10 (it is recommended to weigh about 0.1g of tissue and add 1mL of extraction liquid), then centrifuge at 10000g, 4°C for 20 minutes, take the supernatant and keep it on ice for testing.
2. Bacteria, fungi: Homogenize on ice at a ratio of cell number (104 cells) to extraction liquid volume (mL) of 500~1000:1 (it is recommended to add 1mL of extraction liquid to 5 million cells), ultrasonicate to break the cells (power 300W, ultrasonic for 3 seconds, interval 7 seconds, total time 3 minutes); then centrifuge at 10000g, 4°C for 20 minutes, take the supernatant and keep it on ice for testing.
3. Serum or culture medium: Directly determine.

Determination Operation Table:

	Blank tube	Determination tube
Sample (μL)		500
Extraction liquid (μL)	500	
Reagent one (μL)	400	400
Mix well, water bath at 37°C for 20 minutes		
Reagent two (μL)	300	300

Reagent three (μL)	300	300
Mix well, water bath at 37°C for 20 minutes		
Reagent four (μL)	500	500
Mix well, centrifuge at 8000g, 25°C for 5 minutes, carefully take 1mL of the upper aqueous phase, 1mL glass cuvette, adjust zero with distilled water, determine A530. ΔA = A Determination - A Blank, only one blank tube is needed.		

Calculation Formula for Superoxide Anion Content:

Standard curve: y = 0.0242x - 0.0027, R2 = 0.9980

1. Tissue:

(1) Calculated according to sample mass

Superoxide anion content (nmol/g fresh weight) = (ΔA + 0.0027) ÷ 0.0242 × V total / (V sample / V sample total × W) × 2 = 148.76 × (ΔA + 0.0027) ÷ W

Superoxide anion production rate (nmol/g·min) = 148.76 × (ΔA + 0.0027) ÷ W ÷ T = 7.44 × (ΔA + 0.0027) ÷ W

(2) Calculated according to protein concentration

Superoxide anion content (nmol/mg prot) = (ΔA + 0.0027) ÷ 0.0242 × V total / (V sample × Cpr) × 2

= 148.76 × (ΔA + 0.0027) ÷ Cpr

Superoxide anion production rate (nmol/mg prot·min) = 148.76 × (ΔA + 0.0027) ÷ Cpr ÷ T

= 7.44 × (ΔA + 0.0027) ÷ Cpr

2. Bacteria, fungi:

Superoxide anion content (nmol/104 cell) = (ΔA + 0.0027) ÷ 0.0242 × V total / (V sample / V sample total × cell number) × 2

= 148.76 × (ΔA + 0.0027) ÷ cell number

Superoxide anion production rate (nmol/104 cell·min) = 148.76 × (ΔA + 0.0027) ÷ cell number ÷ T

= 7.44 × (ΔA + 0.0027) ÷ cell number

3. Serum or culture medium:

Superoxide anion content (nmol/mL) = (ΔA + 0.0027) ÷ 0.0242 × V total / V sample × 2

= 148.76 × (ΔA + 0.0027)

Superoxide anion production rate (nmol/mL·min) = 148.76 × (ΔA + 0.0027) ÷ T

= 7.44 × (ΔA + 0.0027)

V sample total: Volume of extraction liquid added, 1 mL; V total: Total reaction volume, 0.9 mL; V sample: Sample volume in the reaction, 0.5 mL; Cpr: Sample protein concentration, mg/mL; W: Sample mass, g; T: Reaction time, 20 min; 2: Two molecules of O_2^- participate in the reaction to generate one molecule of NO_2^-.

Precautions:

1. If the absorbance value is greater than 2, appropriately dilute the sample before re-determination, and remember to multiply by the dilution factor in the calculation formula.
2. After the sample is prepared, proceed with the determination immediately, do not store the sample at low temperatures for a long time, as it may affect the determination results.
3. Reagent four is toxic to some extent, please take protective measures during operation.

Ascorbic Acid (Ascorbic Acid, AsA) Assay Kit Instructions

Spectrophotometric Method 50 Tubes/48 Samples

Note: Select 2-3 samples with expected large differences for pre-determination before the formal test.

Significance of Determination:
AsA, also known as Vitamin C, serves as a coenzyme, free radical scavenger, electron donor/acceptor, and substrate for the biosynthesis of oxalate and tartrate. As the most important antioxidant in plant cells, AsA plays a crucial role in protecting chloroplasts from oxidative damage and is one of the important indicators for measuring the quality of agricultural products.
Principle of Determination:
In acetic acid solution, ascorbic acid reacts with guaiacol blue B to form a yellow oxalate-2-hydroxybutyrolactone derivative, and the absorbance is measured at the maximum absorption wavelength of 420 nm.
Instruments and Supplies to be Prepared:
Mortar and pestle, ice, low-temperature centrifuge, UV spectrophotometer, 1mL glass cuvette, adjustable pipette, and distilled water.
Composition and Preparation of Reagents:
Extraction liquid: Liquid 60mL × 1 bottle, store at 4°C.
Reagent one: Liquid 4mL × 1 bottle, store at 4°C.
Reagent two: Liquid 6mL × 1 bottle, store at 4°C.
Reagent three: Powder × 2 bottles (brown), store at 4°C in the dark. Prepare just before use, add 7 mL of distilled water to each bottle and dissolve thoroughly, unused reagent can be stored at 4°C for up to 3 days. (**Note: The trace amount of powder may not be visible to the naked eye, normal dissolution is sufficient.**)
AsA Extraction from Samples:
1. Tissue: Homogenize on ice at a ratio of tissue mass (g) to extraction liquid volume (mL) of 1:5~10 (it is recommended to weigh about 0.1g of tissue and add 1mL of extraction liquid). Centrifuge at 8000g, 4°C for 20 minutes, take the supernatant and keep it on ice for testing.
2. Bacteria, fungi: Homogenize on ice at a ratio of cell number (104 cells) to extraction liquid volume (mL) of 500~1000:1 (it is recommended to add 1mL of extraction liquid to 5 million cells), ultrasonicate to break the cells (power 300W, ultrasonic for 3 seconds, interval 7 seconds, total time 3 minutes); centrifuge at 8000g, 4°C for 20 minutes, take the supernatant and keep it on ice for testing.
3. Serum and other liquids: Directly determine.
AsA Determination Operation:
1. Preheat the spectrophotometer for 30 minutes, set the wavelength to 420 nm, and zero with distilled water.
2. Add the following reagents to an EP tube:

Reagent Name (μL)	Test Tube	Blank Tube

Sample	200	
Extraction liquid		200
Reagent one	60	60
Reagent two	100	100
Reagent three	240	240
Water	1400	1400

Mix well, let stand at 25°C for 20 minutes, take 1mL and add to a 1mL glass cuvette, measure the absorbance of each tube at 420 nm. ΔA = A Test - A Blank.

Note: Only one blank tube needs to be measured.

AsA Content Calculation Formula:

Standard curve y = 0.0088x - 0.018, R2 = 0.9978

(1) Calculate AsA by protein concentration (μg/mg prot) = (ΔA + 0.018) ÷ 0.0088 × V sample ÷ (Cpr × V sample)

= 113.63 × (ΔA + 0.018) ÷ Cpr

(2) Calculate AsA by sample mass (μg/g fresh weight) = (ΔA + 0.018) ÷ 0.0088 × V sample ÷ (W × V sample ÷ V total sample)

= 113.63 × (ΔA + 0.018) ÷ W

(3) Calculate AsA by cell number (μg/104 cells) = (ΔA + 0.018) ÷ 0.0088 × V sample ÷ (cell number × V sample ÷ V total sample)

= 113.63 × (ΔA + 0.018) ÷ cell number

(4) Calculate AsA by liquid volume (μg/mL) = (ΔA + 0.018) ÷ 0.0088

= 113.63 × (ΔA + 0.018)

V sample: Volume of sample added, 0.2mL; V total sample: Volume of extraction liquid added, 1.0 mL; Cpr: Protein concentration in the supernatant, mg/mL; W: Sample mass (g).

Precautions:

1. Reagent three should be prepared and used immediately, prepared reagent can be stored at 4°C and used up within 3 days.

Reduced Glutathione (GSH) Assay Kit Instructions

Spectrophotometric Method 50 Tubes/48 Samples

Note: Select 2-3 samples with expected large differences for pre-determination before the formal test.

Significance of Determination:
GSH is the most important antioxidant thiol substance in cells and plays a crucial role in antioxidant activity, protection of protein thiol groups, and transmembrane transport of amino acids. The ratio of reduced to oxidized form (GSH/GSSG) is a primary dynamic indicator of the cellular redox state. Therefore, determining the intracellular content of GSH and GSSG, as well as the GSH/GSSG ratio, can well reflect the redox state of the cells.
Principle of Determination:
DTNB reacts with GSH to form a complex with a characteristic absorption peak at 412nm; its absorbance is directly proportional to the GSH content.
Instruments and Supplies to be Prepared:
Visible spectrophotometer, low-temperature centrifuge, water bath, adjustable pipette, 1mL glass cuvette, and distilled water.
Composition and Preparation of Reagents:
Reagent one: Liquid 60mL × 1 bottle, store at 4°C.
Reagent two: Liquid 42mL × 1 bottle, store at 4°C.
Reagent three: Liquid 12mL × 1 bottle, store at 4°C in the dark.
Crude Enzyme Extraction:
1. Tissue: Homogenize on ice at a ratio of tissue mass (g) to reagent one volume (mL) of 1:5~10 (it is recommended to weigh about 0.1g of tissue and add 1mL of reagent one). Centrifuge at 8000g, 4°C for 10 minutes, take the supernatant and keep it on ice for testing.
2. Bacteria, fungi: Homogenize on ice at a ratio of cell number (104 cells) to reagent one volume (mL) of 500~1000:1 (it is recommended to add 1mL of reagent one to 5 million cells), ultrasonicate to break the cells (power 300W, ultrasonic for 3 seconds, interval 7 seconds, total time 3 minutes); then centrifuge at 8000g, 4°C, for 10 minutes, take the supernatant and keep it on ice for testing.
3. Serum and other liquids: Directly determine.
GSH Determination Operation:
1. Preheat the spectrophotometer for 30 minutes, set the wavelength to 412 nm, and zero with distilled water.
2. Preheat reagent two at 37°C for 10 minutes.
3. Blank tube: Take a 1mL glass cuvette, add 100μL of distilled water, 700μL of reagent two, and 200μL of reagent three in sequence, mix well, let stand for 2 minutes, then measure the absorbance A1 at 412 nm.
4. Test tube: Take a 1mL glass cuvette, add 100μL of supernatant, 700μL of reagent two, and 200μL of reagent three in sequence, mix well, let stand for 2 minutes, then measure the absorbance A2 at 412 nm.
Note: Only one blank tube needs to be measured.

GSH Content Calculation Formula:

GSH Standard Curve Formula: y = 1.5x (x is the GSH concentration, μmol/mL; y is the absorbance value)

GSH Calculation:

(1) Calculate GSH by protein concentration (μmol/mg prot) = (A2 - A1) ÷ 1.5 × V sample ÷ (V sample × Cpr) = 0.667 × (A2 - A1) ÷ Cpr

(2) Calculate GSH by sample fresh weight (μmol/g fresh weight) = (A2 - A1) ÷ 1.5 × V sample ÷ (V sample ÷ V total sample × W) = 0.667 × (A2 - A1) ÷ W

(3) Calculate GSH by cell number (μmol/104 cells) = (A2 - A1) ÷ 1.5 × V sample ÷ (V sample ÷ V total sample × cell number) = 0.667 × (A2 - A1) ÷ cell number

(4) Calculate GSH by liquid volume (μmol/mL) = (A2 - A1) ÷ 1.5 × V sample ÷ V sample = 0.667 × (A2 - A1)

V total sample: Total volume of supernatant, 1 mL; V sample: Volume of supernatant added to the reaction system, 100μL = 0.1 mL; W: Sample mass, g; Cpr: Protein concentration in the supernatant, mg/mL.

Precautions:

1. Reagent one contains a protein precipitant, so the supernatant cannot be used for protein concentration determination.
2. The minimum detection limit is 0.01 mmol/L.

Peroxidase (Peroxidase, POD) Assay Kit Instructions

Spectrophotometric Method 50 Tubes/48 Samples

Ensure to take 2-3 samples with expected large differences for pre-determination before the formal test.

Significance of Determination:

POD (EC 1.11.1.7) is widely present in animals, plants, microorganisms, and cultured cells. It can catalyze hydrogen peroxide to oxidize phenolic and amine compounds, playing a dual role in eliminating the toxicity of hydrogen peroxide and phenolic and amine compounds.

Principle of Determination:

POD catalyzes the oxidation of specific substrates by H_2O_2, which has characteristic light absorption at 470nm.

Instruments and Supplies to be Prepared:

Visible spectrophotometer, desktop centrifuge, adjustable pipette, 1mL glass cuvette, mortar and pestle, ice, and distilled water.

Composition of Reagents:

Extraction liquid: Liquid 60mL × 1 bottle, store at 4°C;

Reagent one: Liquid 50mL × 1 bottle, store at 4°C;

Reagent two: Liquid 100μL × 1 vial, store at 4°C;

Reagent three: Liquid 100μL × 1 vial, store at 4°C.

Extraction of Crude Enzyme Solution:

1. Preparation of bacterial, cellular, or tissue samples:

Bacteria or cultured cells: First, collect bacteria or cells into a centrifuge tube, centrifuge and discard the supernatant; according to the ratio of the number of bacteria or cells (104 cells) to the volume of extraction liquid (mL) of 500~1000:1 (it is recommended to add 1mL of extraction liquid to 5 million bacteria or cells), ultrasonicate to break the bacteria or cells (ice bath, power 20% or 200W, ultrasonic for 3 seconds, interval 10 seconds, repeat 30 times); centrifuge at 8000g for 10 minutes at 4°C, take the supernatant, and keep it on ice for testing.

Tissue: According to the ratio of tissue mass (g) to the volume of extraction liquid (mL) of 1:5~10 (it is recommended to weigh about 0.1g of tissue and add 1mL of extraction liquid), homogenize in an ice bath. Centrifuge at 8000g for 10 minutes at 4°C, take the supernatant, and keep it on ice for testing.

2. Serum (plasma) samples: Directly detect.

Determination Steps and Reagent Addition Table:

1. Preheat the spectrophotometer for more than 30 minutes, adjust the wavelength to 470nm, and zero with distilled water.

2. Preparation of working solution: Immediately before use, mix reagent one, reagent two, and reagent three in the ratio of 2.6 (mL): 1.5 (μL): 1 (μL); preheat at 37°C (mammals) or 25°C (other species) for more than 10 minutes; prepare and use immediately.

3. Add 50μL of sample and 950μL of working solution to a 1mL glass cuvette, mix well, and record the absorbance A1 at 470nm after 1 minute and the absorbance A2 after 2

minutes (the time interval between A2 and A1 is 1 minute). Calculate ΔA = A2 - A1.

Notes:
1. It is recommended to take 2-3 samples for pre-determination. After mixing the sample with the working solution, it quickly changes from orange-yellow to reddish-brown, and the A1 value will be very large (>1), and it may be that A2-A1 appears as a negative value, indicating that the enzyme activity is high. The supernatant of the sample extraction can be diluted 10-20 times with extraction liquid or distilled water and re-tested, 10μL supernatant + 90μL extraction liquid or distilled water is a 10-fold dilution. Multiply the result by the corresponding dilution factor.
2. If the A1 value is very small (<0.1), and ΔA is very small (<0.005), and there is no color development after mixing the sample and the working solution for a while, it indicates that the enzyme activity is low. The reaction time can be extended to 10min, 20min, or 30min, and the actual reaction time is substituted into the calculation formula. ③Due to the issues of the samples themselves, the POD of different species varies greatly. Some species have high activity (changing to reddish-brown quickly after adding the working solution); some species have low activity (no color change within 5 minutes after adding the working solution), and the absorbance value only changes to the third decimal place.
POD Activity Calculation:
1. Serum (plasma) POD activity
Unit definition: A change of A470 of 0.01 per mL of serum (plasma) in each mL of reaction system per minute is defined as one enzyme activity unit.
Calculation formula: POD (U/mL) = ΔA × V_total / V_sample / 0.01 / T = 2000 × ΔA
2. Tissue, bacterial, or cellular POD activity
(1) Calculated by sample protein concentration
Unit definition: A change of A470 of 0.01 per mg of tissue protein in each mL of reaction system per minute is defined as one enzyme activity unit.
POD (U/mg prot) = ΔA × V_total / (V_sample × Cpr) / 0.01 / T = 2000 × ΔA / Cpr
(2) Calculated by sample fresh weight
Unit definition: A change of A470 of 0.01 per g of tissue in each mL of reaction system per minute is defined as one enzyme activity unit.
POD (U/g fresh weight) = ΔA × V_total / (W × V_sample / V_total sample) / 0.01 / T = 2000 × ΔA / W
(3) Calculated by bacterial or cellular density
Unit definition: A change of A470 of 0.01 per 10,000 bacteria or cells in each mL of reaction system per minute is defined as one enzyme activity unit.
POD (U/104 cells) = ΔA × V_total / (500 × V_sample / V_total sample) / 0.01 / T = 4 × ΔA
V_total: Total volume of the reaction system, 1mL; V_sample: Volume of sample added, 0.05mL; V_total sample: Volume of extraction liquid added, 1 mL; T: Reaction time, 1 min; Cpr: Sample protein concentration, mg/mL; W: Sample mass.

Catalase (Catalase, CAT) Assay Kit Instructions

Spectrophotometric Method 50 Tubes/48 Samples

Ensure to take 2-3 samples with expected large differences for pre-determination before the formal test.**

Significance of Determination:
CAT (EC 1.11.1.6) is widely present in animals, plants, microorganisms, and cultured cells. It is the most important enzyme for H_2O_2 clearance and plays a crucial role in the reactive oxygen species (ROS) elimination system.
Principle of Determination:
H_2O_2 has a characteristic absorption peak at 240nm. CAT can decompose H_2O_2, causing the absorbance of the reaction solution at 240nm to decrease over time. The activity of CAT can be calculated based on the rate of change in absorbance.
Instruments and Supplies to be Prepared:
UV spectrophotometer, desktop centrifuge, adjustable pipette, 1mL quartz cuvette, mortar and pestle, ice, and distilled water.
Composition and Preparation of Reagents:
Extraction liquid: 60mL × 1 bottle, store at 4°C;
Working solution: 60mL × 1 bottle, store at 4°C.
Extraction of Crude Enzyme Solution:
1. Preparation of bacterial, cellular, or tissue samples:
Bacteria or cultured cells: First, collect bacteria or cells into a centrifuge tube, centrifuge and discard the supernatant; according to the ratio of the number of bacteria or cells (104 cells) to the volume of extraction liquid (mL) of 500~1000:1 (it is recommended to add 1mL of extraction liquid to 5 million bacteria or cells), ultrasonicate to break the bacteria or cells (ice bath, power 20% or 200W, ultrasonic for 3 seconds, interval 10 seconds, repeat 30 times); centrifuge at 8000g for 10 minutes at 4°C, take the supernatant, and keep it on ice for testing.
Tissue: According to the ratio of tissue mass (g) to the volume of extraction liquid (mL) of 1:5~10 (it is recommended to weigh about 0.1g of tissue and add 1mL of extraction liquid), homogenize in an ice bath. Centrifuge at 8000g for 10 minutes at 4°C, take the supernatant, and keep it on ice for testing.
2. Serum (plasma) samples: Directly test.
Determination Steps:
1. Preheat the spectrophotometer for more than 30 minutes, set the wavelength to 240nm, and zero with distilled water.
2. Before the test, incubate the CAT detection working solution at 37°C (mammals) or 25°C (other species) in a water bath for 10 minutes.
3. In a 1mL quartz cuvette, add 35μL of sample and 1mL of working solution, mix well, immediately measure the initial absorbance A1 at 240nm at room temperature and the absorbance A2 after 1 minute, calculate ΔA = A1 - A2.
Note on Negative Values:
Check if bubbles are produced during the reaction process. Many bubbles indicate high

enzyme activity, which may affect and cause negative values. The sample can be diluted 10 times with extraction liquid and tested again. If the diluted sample or reaction system still shows a small negative value without bubble production, it indicates that the enzyme activity in the sample cannot be detected.

Calculation of CAT Activity:

1. Calculation of CAT activity in serum (plasma):

The unit is defined as the degradation of 1nmol H_2O_2 per milliliter of serum (plasma) per minute.

CAT (nmol/min/mL) = [ΔA × V_total / (ε × d) × 10^9] ÷ V_sample ÷ T = 678 × ΔA

2. Calculation of CAT activity in tissues, bacteria, or cells:

(1) Calculated by sample protein concentration:

The unit is defined as the degradation of 1nmol H_2O_2 per milligram of tissue protein per minute.

CAT (nmol/min/mg prot) = [ΔA × V_total / (ε × d) × 10^9] ÷ (V_sample × Cpr) ÷ T = 678 × ΔA ÷ Cpr

(2) Calculated by sample fresh weight:

The unit is defined as the degradation of 1nmol H_2O_2 per gram of tissue per minute.

CAT (nmol/min/g fresh weight) = [ΔA × V_total / (ε × d) × 10^9] ÷ (W × V_sample ÷ V_total sample) ÷ T = 678 × ΔA ÷ W

(3) Calculated by bacterial or cellular density:

The unit is defined as the degradation of 1nmol H_2O_2 per 10,000 bacteria or cells per minute.

CAT (nmol/min/104 cells) = [ΔA × V_total / (ε × d) × 10^9] ÷ (500 × V_sample ÷ V_total sample) ÷ T = 1.356 × ΔA

V_total: Total volume of the reaction system, 1.035 × 10^-3 L;

ε: Molar extinction coefficient of H_2O_2, 4.36 × 10^4 L / mol / cm;

d: Cuvette light path, 1 cm;

V_sample: Volume of sample added, 0.035 mL;

V_total sample: Volume of extraction liquid added, 1 mL;

T: Reaction time, 1 min;

W: Sample mass, g;

Cpr: Sample protein concentration, mg/mL;

500: Total number of cells or bacteria, 5 million.

Superoxide Dismutase (SOD) Assay Kit Instructions (NBT Method)

Spectrophotometric Method 50 Tubes/48 Samples

Ensure to take 2-3 samples with expected large differences for pre-determination before the formal test.**

Significance of Determination:
SOD (EC 1.15.1.1) is widely present in animals, plants, microorganisms, and cultured cells. It catalyzes the dismutation of superoxide anions, generating H_2O_2 and O_2. SOD is not only a superoxide anion scavenging enzyme but also a primary H_2O_2 generating enzyme, playing an important role in the biological antioxidant system.

Principle of Determination:
The xanthine and xanthine oxidase reaction system produces superoxide anions ($O_2^-.$), which can reduce nitro blue tetrazolium to form blue formazan, the latter having absorption at 560nm; SOD can clear $O_2^-.$, thereby inhibiting the formation of formazan; the deeper the blue color of the reaction liquid, the lower the SOD activity, and vice versa.

Instruments and Supplies to be Prepared:
Visible spectrophotometer, desktop centrifuge, adjustable pipette, 1mL glass cuvette, mortar and pestle, ice, and distilled water.

Composition and Preparation of Reagents:
Extraction liquid: Liquid 60mL × 1 bottle, store at 4°C;
Reagent one: Liquid 15mL × 1 bottle, store at 4°C;
Reagent two: Liquid 350μL × 1 vial, store at 4°C;
Reagent three: Liquid 10mL × 1 bottle, store at 4°C;
Reagent four: Powder × 2 bottles, store at 4°C.

Extraction of Crude Enzyme Solution:
1. Preparation of bacterial, cellular, or tissue samples:
Bacteria or cultured cells: First, collect bacteria or cells into a centrifuge tube, centrifuge and discard the supernatant; according to the ratio of the number of bacteria or cells (104 cells) to the volume of extraction liquid (mL) of 500~1000:1 (it is recommended to add 1mL of extraction liquid to 5 million bacteria or cells), ultrasonicate to break the bacteria or cells (ice bath, power 20% or 200W, ultrasonic for 3 seconds, interval 10 seconds, repeat 30 times); centrifuge at 8000g for 10 minutes at 4°C, take the supernatant, and keep it on ice for testing.
Tissue: According to the ratio of tissue mass (g) to the volume of extraction liquid (mL) of 1:5~10 (it is recommended to weigh about 0.1g of tissue and add 1mL of extraction liquid), homogenize in an ice bath. Centrifuge at 8000g for 10 minutes at 4°C, take the supernatant, and keep it on ice for testing.
2. Serum (plasma) samples: Directly test.

Determination Steps:
1. Preheat the spectrophotometer for more than 30 minutes, set the wavelength to 560nm, and zero with distilled water.
2. Dilute reagent two with distilled water twice, prepare as much as needed. (Dilute reagent two with distilled water at a 1:1 ratio)

3. Dissolve one bottle of reagent four with 5mL of distilled water (use within one week after dissolution), then dilute with distilled water four times, prepare as much as needed. (Dilute reagent four with distilled water at a 1:3 ratio)
4. Before testing, incubate reagents one, three, and four in a water bath at 37°C (mammals) or 25°C (other species) for more than 5 minutes.
5. Sample determination (add the following reagents in order in an EP tube):

Reagent Name (μL)	Test Tube	Control Tube
Reagent one	240	240
Reagent two	6	6
Sample	90	
Distilled water		90
Reagent three	180	180
Reagent four	510	510

Mix well, let stand at room temperature for 30 minutes, then add to a 1mL glass cuvette, and measure the absorbance A of each tube at 560nm.

Precautions:

1. Reagent two is an enzyme and should not be frozen. Place it on ice when in use.
2. Only one control tube is needed.
3. If the control tube absorbance is greater than 2, it is recommended to dilute reagent two with distilled water by 7 times before use (10μL of reagent two original liquid + 60μL of distilled water).
4. Why is the test tube greater than the control tube for some samples? What is the range of values for the control tube?

The range for the control tube is 0.8-2. A low control tube absorbance may be due to (1) reagent two or reagent four not being prepared and used immediately; (2) reagents not being added in order; (3) insufficient reaction time, which can be extended (the reaction time of 30min can be extended to 40min). A high control tube absorbance may be due to reagent two not being diluted according to the operating manual.

If the test tube is greater than the control tube, it may be that the impurities in the sample are too large. To reduce the impact of impurities, the sample extraction supernatant is generally diluted 10 times with distilled water or extraction liquid and then measured, which usually allows for normal measurement. Multiply by the corresponding dilution factor in the calculation formula.

SOD Activity Calculation:

1. Calculation of inhibition percentage: Inhibition percentage = (A of control tube - A of test tube) ÷ A of control tube × 100%. Try to keep the sample's inhibition percentage within the 10-90% range. If the calculated inhibition percentage is less than 10% or greater than 90%, it is usually necessary to adjust the sample volume and re-determine. If the measured inhibition percentage is too high, the sample should be diluted appropriately with extraction liquid; if the measured inhibition percentage is too low, a more concentrated sample should be prepared.
2. SOD enzyme activity unit: In the above xanthine oxidase coupled reaction system, when the inhibition percentage is 50%, the SOD enzyme activity in the reaction system is defined as one enzyme activity unit (U/mL).

3. SOD enzyme activity calculation:

(1) Serum (plasma) SOD activity (U/mL) = [Inhibition percentage ÷ (1 - Inhibition percentage) × V_total] ÷ V_sample = 11.4 × Inhibition percentage ÷ (1 - Inhibition percentage)

(2) Tissue, bacteria, or cultured cell SOD activity calculation:

a. Calculate SOD activity by sample protein concentration (U/mg prot) = [Inhibition percentage ÷ (1 - Inhibition percentage) × V_total] ÷ (V_sample × Cpr) = 11.4 × Inhibition percentage ÷ (1 - Inhibition percentage) ÷ Cpr

This requires additional measurement and it is recommended to use the BCA protein assay kit from our company.

b. Calculate SOD activity by sample fresh weight (U/g fresh weight) = [Inhibition percentage ÷ (1 - Inhibition percentage) × V_total] ÷ (W × V_sample ÷ V_total sample) = 11.4 × Inhibition percentage ÷ (1 - Inhibition percentage) ÷ W

c. Calculate SOD activity by bacterial or cellular count (U/104 cells) = [Inhibition percentage ÷ (1 - Inhibition percentage) × V_total] ÷ (500 × V_sample ÷ V_total sample) = 0.0228 × Inhibition percentage ÷ (1 - Inhibition percentage)

V_total: Total volume of the reaction system, 1.026mL; V_sample: Volume of sample added to the reaction system, 0.09mL; V_total sample: Volume of extraction liquid added, 1 mL; Cpr: Sample protein concentration, mg/mL; W: Sample mass, g; 500: Total number of cells or bacteria, 5 million.

Ascorbate Peroxidase (APX) Activity Assay Kit Instructions

Spectrophotometric Method 50 Tubes/48 Samples

Note: Select 2-3 samples with expected large differences for pre-determination before the formal test.

Significance of Determination:

APX is one of the important antioxidant enzymes in plants for scavenging reactive oxygen species and is also one of the key enzymes in ascorbic acid metabolism. APX has multiple isoenzymes, which are located in chloroplasts, cytoplasm, mitochondria, peroxisomes, and glyoxysomes, as well as on the peroxisome and thylakoid membranes. APX catalyzes the oxidation of AsA by H_2O_2 and is the main consumer of AsA in plants. The activity of APX directly affects the content of AsA, and there is a certain negative correlation between APX and AsA.

Principle of Determination:

APX catalyzes the oxidation of AsA by H_2O_2. The activity of APX is calculated by measuring the oxidation rate of AsA.

Instruments and Supplies to be Prepared:

Low-temperature centrifuge, UV spectrophotometer, 1mL quartz cuvette, pipette gun, mortar and pestle, ice, and distilled water.

Composition and Preparation of Reagents:

Reagent one: Liquid 90mL × 1 bottle, store at 4°C.

Reagent two: Powder × 2 bottles, store at 4°C. Add 2.5 mL of distilled water to dissolve completely before use (store at 4°C and use within 3 days after dissolution).

Reagent three: Liquid 5mL × 1 vial, store at 4°C.

Extraction of Crude Enzyme Solution:

Homogenize on ice at a ratio of tissue mass (g) to reagent one volume (mL) of 1:5~10 (it is recommended to weigh about 0.1g of tissue and add 1mL of reagent one). Centrifuge at 13000g for 20min at 4°C, take the supernatant and keep it on ice for testing.

Determination:

1. Preheat the spectrophotometer for 30 min, set the wavelength to 290nm, and zero with distilled water.
2. Preheat reagent one at 25°C for 30min.
3. In a 1mL quartz cuvette, add 100μL of supernatant, 700μL of preheated reagent one, 100μL of reagent two, and 100μL of reagent three in sequence. Mix quickly and measure the light absorption A1 at 10s and A2 at 130s at 290nm, ΔA = A1 - A2.

APX Activity Calculation Formula:

(1) Calculated by sample protein concentration

Definition of activity unit: The oxidation of 1nmol AsA per milligram of protein per minute is defined as one enzyme activity unit.

APX (nmol/min/mg prot) = ΔA ÷ (ε × d) × V_total × 10^9 ÷ (Cpr × V_sample) ÷ T

= 1786 × ΔA ÷ Cpr

(2) Calculated by sample mass

Definition of activity unit: The oxidation of 1nmol AsA per gram of tissue per minute is defined as one enzyme activity unit.

APX (nmol/min/g fresh weight) = $\Delta A \div (\varepsilon \times d) \times V\_total \times 10^9 \div (W \times V\_sample \div V\_total\_sample) \div T$

$= 1786 \times \Delta A \div W$

ε: The molar absorption coefficient of AsA at 290nm is 2.8×10^3 L/mol/cm;

d: Cuvette light path (cm), 1 cm;

V_total: Total volume of the reaction system (L), 1000μL = 1×10^{-3} L;

10^9: 1 mol = 1×10^9 nmol;

V_sample: Volume of supernatant added to the reaction system (mL), 100μL = 0.1 mL;

V_total_sample: Volume of extraction liquid added, 1 mL;

Cpr: Protein concentration in the supernatant (mg/mL), which needs to be measured separately, it is recommended to use the BCA protein assay kit from our company;

W: Sample mass, g;

T: Catalytic reaction time (min), 2 min.

Glutathione Peroxidase (GSH-Px) Instruction Manual

Spectrophotometric Method 50 Tubes/48 Samples

Note: Select 2-3 samples with expected large differences for pre-determination before the formal test.

Significance of Determination:
GSH-Px is one of the main enzymes that catalyze the oxidation of reduced glutathione (GSH) in the glutathione redox cycle. GSH-Px not only specifically catalyzes the reaction of reduced glutathione with ROS to produce oxidized glutathione (GSSG), thereby protecting the biomembrane from ROS damage and maintaining normal cell function; it also has the ability to protect the liver, enhance immune function, antagonize the harm of harmful metal ions to the body, and increase the body's radiation resistance.
Principle of Determination:
GSH-Px catalyzes the oxidation of GSH by organic peroxides to produce GSSG; glutathione reductase (GR) catalyzes the reduction of GSSG by NADPH, regenerating GSH, while NADPH is oxidized to NADP+; NADPH has a characteristic absorption peak at 340 nm, while NADP+ does not; the activity of GSH-Px is calculated by measuring the rate of decrease in light absorption at 340 nm.
Instruments and Supplies to be Prepared:
UV spectrophotometer, low-temperature centrifuge, water bath, adjustable pipette, 1mL quartz cuvette, and distilled water.
Composition and Configuration of Reagents:
Reagent one: Liquid 100mL × 1 bottle, store at room temperature.
Reagent two: Powder × 2 bottles, store at 4°C.
Reagent three: Liquid 10μL × 2 vials, store at -20°C.
Reagent four: Liquid 200μL × 1 bottle, store at 4°C.
Extraction of Crude Enzyme Solution:
1. Tissue: Homogenize on ice at a ratio of tissue mass (g) to reagent one volume (mL) of 1:5~10 (it is recommended to weigh about 0.1g of tissue and add 1mL of reagent one). Centrifuge at 8000g for 10min at 4°C, take the supernatant and keep it on ice for testing.
2. Bacteria, fungi: Homogenize on ice at a ratio of cell number (104 cells) to reagent one volume (mL) of 500~1000:1 (it is recommended to add 1mL of reagent one to 5 million cells), ultrasonicate to break the cells (power 300W, ultrasonic for 3 seconds, interval 7 seconds, total time 3 minutes); then centrifuge at 8000g, 4°C, for 10 minutes, take the supernatant and keep it on ice for testing.
3. Serum and other liquids: Directly determine.
Determination Operation:
1. Preheat the spectrophotometer for 30 minutes, set the wavelength to 340 nm, and zero with distilled water.
2. Preheat the mixed reagents in a water bath at 25°C or 37°C (for mammals) for 30 minutes.
3. Preparation of mixed reagents: Immediately before use, take one bottle of reagent two and add to 20 mL of reagent one, shake well to dissolve, then take a small amount of the

mixture and add it to one vial of reagent three, mix well before transferring it back to reagent two, mix and wait for use. (Note: Must be prepared and used immediately, finish on the same day)
4. Dilution of reagent four: Take 21.5μL of reagent four and add to 5 mL of water, mix well, prepare immediately before use, and use the prepared reagent on the same day.
5. Determination tube: In a 1mL quartz cuvette, add 100μL of supernatant, 800μL of preheated mixed reagent, and 100μL of diluted reagent four in sequence, mix quickly and measure the absorbance at 340nm at the 30th and 210th second, recorded as A1 and A2, respectively, $\Delta A = A1 - A2$.

Calculation Formula:

(1). Calculated by protein concentration
Definition of GSH-Px activity unit: At a certain temperature, the catalytic oxidation of 1nmol NADPH per mg of protein per minute is defined as one enzyme activity unit.
GSH-Px (nmol/min/mg prot) = $[\Delta A \div \varepsilon \div d \times V\_total \times 10^9] \div (Cpr \times V\_sample) \div T$
$= 536 \times \Delta A \div Cpr$
(2). Calculated by sample mass
Definition of GSH-Px activity unit: At a certain temperature, the catalytic oxidation of 1nmol NADPH per g of sample per minute is defined as one enzyme activity unit.
GSH-Px (nmol/min/g) = $[\Delta A \div \varepsilon \div d \times V\_total \times 10^9] \div (W \times V\_sample \div V\_total\_sample) \div T$
$= 536 \times \Delta A \div W$
(3). Calculated by cell number
Definition of activity unit: At a certain temperature, the catalytic oxidation of 1nmol NADPH per 10^4 cells per minute is defined as one enzyme activity unit.
GSH-Px (nmol/min/10^4 cell) = $[\Delta A \div \varepsilon \div d \times V\_total \times 10^9] \div (\text{cell number} \times V\_sample \div V\_total\_sample) \div T$
$= 536 \times \Delta A \div \text{cell number}$
(4). Calculated by liquid volume
Definition of activity unit: At a certain temperature, the catalytic oxidation of 1nmol NADPH per mL of liquid per minute is defined as one enzyme activity unit.
GSH-Px (nmol/min/mL) = $[\Delta A \div \varepsilon \div d \times V\_total \times 10^9] \div V\_sample \div T$
$= 536 \times \Delta A$
ε: The molar extinction coefficient of NADPH is 6.22×10^3 L/mol/cm;
V_total: Total volume of the reaction system, 1000μL = 0.001 L;
10^6: 1 mol = 1×10^6 μmol;
Cpr: Protein concentration in the supernatant (mg/mL);
V_sample: Volume of supernatant added to the reaction system, 100μL = 0.1 mL;
V_total_sample: Volume of extraction liquid added, 1 mL;
W: Sample mass, g;
T: Reaction time, 3 min.

Precautions:

1. Sample processing and other procedures must be carried out on ice and enzyme activity must be determined on the same day;
2. Mixed reagents and substrate solutions must be prepared immediately before use,

prepared on ice, and used up on the same day;

3. The determination process must be carried out quickly;

4. When determining the activity of GSH-Px in cells, the number of cells must be between 3 million and 5 million. When extracting GSH-Px from cells, grinding or ultrasonic treatment can be done after adding reagent one. Cell lysis fluid must not be used to treat cells.
